# Association between potentially inappropriate medications and 30-day outcomes among hospitalized older adults with reduced physiologic reserve: a retrospective cohort study

**DOI:** 10.3389/fphar.2026.1813958

**Published:** 2026-05-20

**Authors:** Zhen Zeng, Fusong Peng, Peng Liang

**Affiliations:** Department of Geriatrics, Chuiyangliu Hospital Affiliated with Tsinghua University, Beijing, China

**Keywords:** older adults, patient readmission, polypharmacy, potentially inappropriate medications, propensity score matching, proton pump inhibitors

## Abstract

**Objectives:**

To investigate the independent effect of potentially inappropriate medications (PIMs) on 30-day adverse post-discharge outcomes (unplanned readmissions or emergency department [ED] visits) among hospitalized older adults after strictly controlling for confounders, such as polypharmacy and psychological comorbidities, and to identify high-risk phenotype in patients with reduced physiologic reserve and specific drug classes associated with adverse outcomes.

**Methods:**

This retrospective cohort study included 1,264 older patients (aged ≥65 years). PIMs were identified at discharge. Multivariable Cox regression and 1:1 propensity score matching (PSM, caliper 0.02) were used to adjust for confounders, including medication count and psychological comorbidities. Sensitivity, subgroup, drug-specific, and E-value analyses were performed to assess risk heterogeneity and robustness.

**Results:**

Overall, 41.8% of the patients (n = 529) were exposed to PIMs. PIM status was not significantly associated with the 30-day composite outcome (unplanned readmission or ED visits) in either the total population (n = 1,264; adjusted hazard ratio [AHR] 1.22, 95% confidence interval [CI] 0.92–1.62, *P* = 0.16) or the PSM cohort (n = 776; AHR 1.12, 95% CI 0.81–1.54, *P* = 0.49). However, subgroup analysis revealed significant risk elevation in critically ill patients (AHR 1.84, 95% CI 1.14–2.99), although the interaction was not statistically significant (*P* = 0.07). Additionally, inappropriate proton pump inhibitor (PPI) use was associated with increased risk (AHR 1.39, 95% CI 1.02–1.89).

**Conclusion:**

After controlling for polypharmacy, PIM was not a uniform predictor of the 30-day composite outcome, but it served as a marker of clinical complexity. However, it became a significant marker of risk in patients with reduced physiologic reserve and in those with inappropriate PPI use. Clinical interventions should prioritize these high-risk phenotypes for precision deprescribing.

## 1 Introduction

With global population aging, multimorbidity has become the norm among hospitalized older patients, leading to widespread polypharmacy (Masnoon et al., 2017). Although pharmacotherapy remains the cornerstone of disease management, declines in physiologic reserve and the associated alterations in drug pharmacokinetics and pharmacodynamics render older adults highly susceptible to adverse drug events (ADEs) (Mangoni and Jackson, 2004; Maher et al., 2014). To identify and mitigate these risks, screening tools for potentially inappropriate medications (PIMs), as defined by the 2023 American Geriatric Society (AGS) Beers Criteria (American Geriatric Society, 2023), have been widely implemented worldwide. It is estimated that the prevalence of PIM use among older patients ranges from 30% to 60% (Tian et al., 2023). Despite the promotion of screening tools, whether PIMs independently contribute to clinical endpoints—such as 30-day readmissions—remains a subject of intense debate (Weir et al., 2020; Sombut et al., 2025). Thirty-day post-discharge readmission is not merely a clinical outcome; it has evolved into a critical quality metric for hospital care, directly influencing healthcare reimbursement and penalty mechanisms in many medical systems (Oshima Lee and Emanuel, 2013). Therefore, identifying modifiable risk factors for 30-day readmissions, such as inappropriate prescribing, holds substantial health-economic value.

The independent effect of PIMs on 30-day readmissions remains academically controversial. An early study suggested that newly initiated or continued PIMs at discharge significantly increase the risk of adverse events and readmissions (Weir et al., 2020). In contrast, a recent large-scale study observed no significant association between PIMs and early unplanned readmissions (Sombut et al., 2025). This inconsistency may stem from methodological limitations in controlling for the “sick-user effect” in previous research. Specifically, while most studies adjusted for the Charlson Comorbidity Index (CCI), many failed to adequately consider medication count—a powerful surrogate indicator of disease severity and frailty (Fabbietti et al., 2018; Toh et al., 2023). Psychological comorbidities (e.g., anxiety and depression) are also frequently overlooked, despite patients with psychological comorbidities being high-frequency users of PIMs, such as benzodiazepines (Masumoto et al., 2018). Without rigorously disentangling these confounders, it remains unclear whether readmissions are driven by drug toxicity or underlying clinical complexity.

Another consideration is that previous studies have often treated all PIMs as a homogeneous group, which may dilute the signals of specific high-risk drug classes. For instance, proton pump inhibitors (PPIs) are among the most overprescribed medications in older adults and are widely considered a PIM in the context of polypharmacy. They are included in criteria that identify inappropriate prescriptions for older adults, such as the Beers criteria (American Geriatric Society, 2023) and the STOPP/START criteria (Szoszkiewicz et al., 2024). By altering gastrointestinal pH, PPIs potentially increase the risk of hospital-acquired pneumonia (HAP) and *Clostridioides difficile* infection, acting as “invisible drivers” of short-term readmission (Maideen, 2023). Furthermore, it is possible that the same PIM may be tolerated by a robust older adult but may precipitate readmission in a patient with functional impairment or reduced physiologic reserve (Hilmer et al., 2019).

Physiologic reserve, defined as the fundamental factor underlying an individual’s ability to withstand stressors (Chhetri et al., 2021), is a multidimensional indicator of resilience to biological stresses, indicating one’s ability to maintain homeostasis across organ systems (Hu et al., 2024; Saloner et al., 2022; Whitson et al., 2015). Reduced physiologic reserve lowers the threshold for drug tolerance and increases the risk of PIM-related adverse outcomes in older adults (McLean and Le Couteur, 2004). Therefore, when evaluating the effects of PIMs, characteristics such as drug class and physiologic reserve should be considered. However, as physiologic reserve is a complex construct lacking a single standardized measurement (Whitson et al., 2015), recent structural equation modeling studies have proposed the use of clinical illness severity indices to assess physiologic reserve in real-world settings (Hu et al., 2024).

When evaluating the effects of PIMs on 30-day readmission, few studies have used rigorous propensity score matching (PSM) techniques to explore the interaction between specific drug classes and vulnerable populations while strictly controlling for total medication count and psychological comorbidities. Therefore, this retrospective cohort study applied 1:1 PSM to strictly control for these key confounders. This study aimed to (1) evaluate whether overall PIM use is an independent predictor of 30-day adverse outcomes (readmission or emergency department [ED] visits) among older patients after multivariable adjustment for key clinical and pharmacological confounders and (2) determine whether this risk is driven by specific drug classes (e.g., PPIs) or specific patient phenotypes (e.g., critical illness), thereby providing evidence for precision deprescribing strategies (individualized, data-driven reduction or discontinuation of medications that are no longer beneficial, are inappropriate, or are causing adverse effects or increased healthcare resource utilization) in clinical practice.

## 2 Materials and methods

### 2.1 Study design and population

This retrospective cohort study was conducted using data extracted from the electronic medical record system of Tsinghua University Chuiyangliu Hospital. The study period spanned from 1 January 2024, to 31 December 2025. The study was approved by the Institutional Review Board of Tsinghua University Chuiyangliu Hospital (Ethics Approval No. 2026-008KY).

All patients aged ≥65 years discharged from the geriatric ward during the study period were included in this retrospective cohort study. The exclusion criteria were (1) hospital length of stay (LOS) < 24 h; (2) in-hospital death or transfer to hospice/palliative care; (3) discharge against medical advice (including self-discharge or transfer to rehabilitation facilities); (4) lack of necessary baseline data (e.g., discharge medication records); and (5) planned readmissions (e.g., for chemotherapy or dialysis).

### 2.2 Data collection and variables

#### 2.2.1 Exposure

PIM use was defined according to the 2023 American Geriatrics Society Beers Criteria (American Geriatrics Society, 2023). Discharge medication lists were reviewed for each patient. Patients prescribed at least one medication listed under the “medications to be avoided” or “medications to be avoided in specific disease states” categories were assigned to the PIM group. For PPIs, as only 30-day medication information was available, longer durations of use could not be accurately assessed. Indication-based assessment was therefore considered a feasible alternative, as reported previously in epidemiological studies on PIMs using similar hospital or prescription databases (Ivanova et al., 2018). Moreover, short-term post-discharge studies have shown that evaluating lack of clinical indication is a rigorous and highly relevant proxy for identifying inappropriate prescribing (Blanc et al., 2018). Therefore, aligning with the principle that any drug prescribed without an evidence-based indication constitutes a PIM, PPI use within 30 days without a strong indication was defined as PIM use in the present study. Otherwise, the patients were assigned to the non-PIM group.

#### 2.2.2 Covariates

A comprehensive set of demographic and clinical characteristics was collected. For demographic characteristics, age and sex were recorded. For clinical characteristics, CCI (Charlson et al., 1987), Activities of Daily Living (ADL) score (assessed at discharge), frailty status (Dalleur et al., 2012), cognitive impairment (Kristensen et al., 2018), history of fractures (Masumoto et al., 2018), anxiety/depression (Meng et al., 2022), and critical condition (defined as the presence of at least one of the following during hospitalization: intensive care unit [ICU] admission (Rahman et al., 2003), heart failure [New York Heart Association functional class III or above] (Li et al., 2023), respiratory failure, ventilator use, or use of vasoactive drugs). To evaluate the medication burden, the total number of medications at discharge (Ma et al., 2019) was recorded. Other factors, including hospital LOS, total hospitalization costs, and history of readmission within the previous 6 months (Glans et al., 2020), were also evaluated.

### 2.3 Outcome measures

The primary outcome was a 30-day composite endpoint consisting of unplanned hospital readmission or ED visit within 30 days after discharge. Patients who died within 30 days were excluded from the outcome analysis, as death precludes the occurrence of readmission or ED visit.

### 2.4 Statistical analysis

#### 2.4.1 Software and descriptive statistics

Data cleaning and statistical analyses were performed using Python software (version 3.10) with the scientific computing libraries pandas, scipy, and statsmodels.

#### 2.4.2 Comparison of baseline characteristics

Descriptive statistics were used to summarize the baseline characteristics of the study population. Continuous variables were presented as the median (interquartile range) and were compared using the Mann–Whitney *U* test, as the data exhibited a non-normal distribution (assessed by the Shapiro–Wilk test). Categorical variables were expressed as frequencies and percentages [n (%)] and were compared using the chi-square test or Fisher’s exact test, as appropriate.

#### 2.4.3 Association analysis

To identify factors associated with PIM use at discharge, we performed univariable and multivariable logistic regression analyses, and the results are presented as odds ratios (ORs) and 95% confidence intervals (CIs). To evaluate the association between PIM status and the 30-day composite outcome (unplanned readmission or ED visit), we used Cox proportional-hazards models. Univariable Cox regression was performed first for each covariate, followed by a multivariable-adjusted model incorporating clinically relevant covariates, including age, sex, ADL score, CCI score, total medication count, critical condition, and other baseline comorbidities. Hazard ratios (HRs) and 95% CIs were calculated. The cumulative incidence of the 30-day composite outcome was visualized using Kaplan–Meier survival curves, and differences between the groups were evaluated using the log-rank test.

#### 2.4.4 PSM

To minimize potential selection bias and ensure baseline balance between the PIM and non-PIM groups, 1:1 PSM was conducted using a logistic regression model. As highlighted previously, in the context of pharmacoepidemiological challenges, observational studies are susceptible to confounding by indication, where the factors influencing PIM prescribing (such as multimorbidity) also predict outcomes (Degen and Schottker, 2025). PSM addresses this limitation by conditioning the probability of exposure given the observed covariates, creating a pseudo-randomized cohort. This approach reduces model dependency compared with conventional covariate adjustment, particularly given the high-dimensional nature of the data in the present study, ensuring that the estimated association between PIM and the 30-day composite outcome was less biased by baseline imbalances. The propensity scores were estimated based on all baseline covariates. Matching was performed using the nearest neighbor algorithm with a stringent caliper of 0.02. The quality of matching was assessed by calculating standardized mean differences (SMDs) for all covariates, with an SMD of <0.1 considered indicative of optimal balance. A Love plot was generated to visualize the SMD before and after matching. Multivariable Cox regression was subsequently repeated in the PSM cohort.

#### 2.4.5 Subgroup, drug-specific, and sensitivity analyses

As 1:1 PSM inevitably reduces the effective sample size and may decrease statistical power, we conducted a sensitivity analysis using Inverse Probability of Treatment Weighting (IPTW) with stabilized weights following established methodological guidance (Garrido et al., 2014). The analysis included the overall cohort, and the IPTW-adjusted HR for the 30-day composite outcome was evaluated.

Subgroup analyses were also performed across several clinical strata, including critical condition subgroup (indicating physiologic reserve), age (65 to <80 vs. ≥80 years), sex, ADL score (<60 vs. ≥60), and medication count (<5 vs. ≥5 medications (Costanzo et al., 2024; Nicholson et al., 2024)). As physiologic reserve cannot be measured using a single test, we followed the methodology of Hu et al. (2024) and used critical condition (defined as the presence of at least one of the following during hospitalization: ICU admission, heart failure [New York Heart Association functional class III or above], respiratory failure, ventilator use, or use of vasoactive drugs) as an objective clinical proxy to identify patients experiencing an acute, severe depletion of physiologic reserve. *P* for interaction was calculated using likelihood ratio tests to assess the heterogeneity of the association across subgroups. Specifically, for each subgroup variable, we constructed a separate multivariable Cox proportional-hazards model that included the following terms: (i) PIM status, (ii) the subgroup variable of interest (e.g., critical condition status, age group), and (iii) their product term (PIM × subgroup variable). The statistical significance of the interaction was assessed using the likelihood ratio test, comparing the model with the product term to the model without it. Both models were adjusted for all baseline covariates, excluding the stratification variable itself. Analyses of specific PIM subclasses, including PPIs and benzodiazepines, were also conducted.

Sensitivity analyses were conducted by tightening the PSM caliper to 0.01 to test the robustness of the findings. For significant positive associations (critical condition subgroup and PPI-related PIM), E-values were computed to quantify the minimum strength of unmeasured confounding required to negate the observed effects. All statistical tests were two-sided, and *P* < 0.05 was considered statistically significant.

## 3 Results

### 3.1 Study population and baseline characteristics

**FIGURE 1:**
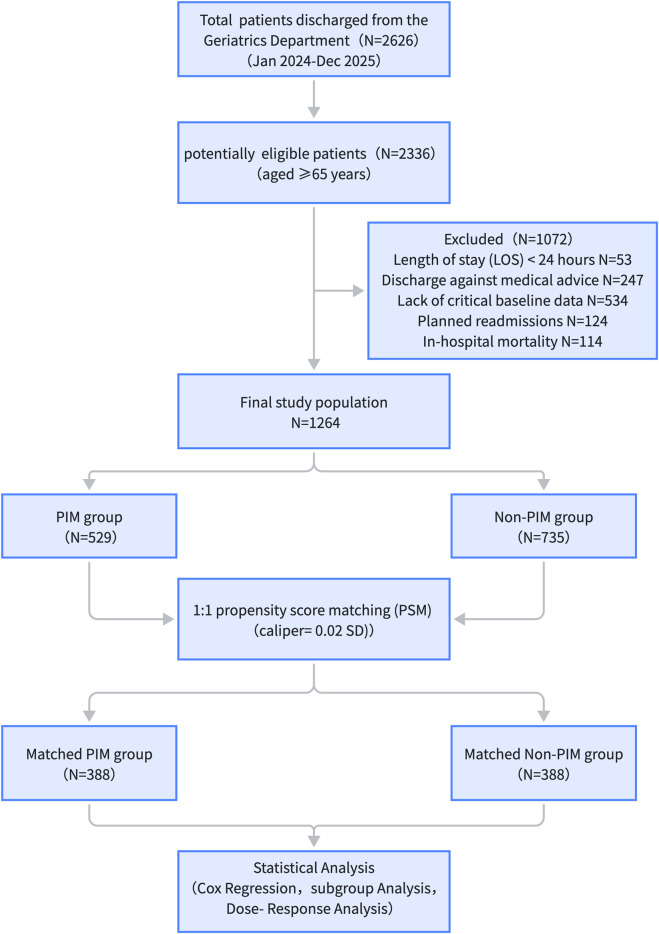
Study flow diagram of patient enrollment and follow-up.

The study cohort comprised 1,264 patients, with 41.8% (n = 529) exposed to PIMs at discharge (Figure 1). The median age was consistent across both groups (non-PIM group: 84.0 [74.0, 88.0] years; PIM group: 84.0 [75.0, 89.0] years). While broad homogeneity was observed regarding sex, functional status (ADL score), and comorbidity burden (CCI score) (all *P* > 0.05), several critical imbalances in clinical complexity were noted. Notably, the PIM group exhibited a substantially higher medication count (median 6.0 vs. 5.0, *P* < 0.001) and a significantly higher prevalence of anxiety/depression (13.8% vs. 4.6%, *P* < 0.001) than the non-PIM group. Conversely, frailty was more frequent in the non-PIM group (9.1% vs. 4.9%, *P* = 0.01). Given these baseline disparities in medication count and psychological status, PSM was performed to determine the effect of PIMs independent from these confounding factors (Table 1).

**TABLE 1:** Baseline characteristics of patients categorized by PIM status.

Characteristic	Non-PIM (N = 735)	PIM (N = 529)	*P* value
Age, years	84.0 (74.0, 88.0)	84.0 (75.0, 89.0)	0.59
Sex (female), n (%)	392 (53.3)	298 (56.3)	0.32
ADL score	65.0 (40.0, 90.0)	65.0 (40.0, 85.0)	0.30
CCI	2.0 (1.0, 3.0)	2.0 (1.0, 3.0)	0.35
Critical condition, n (%)	194 (26.4)	135 (25.5)	0.78
Hospital length of stay, days	6.0 (5.0, 8.0)	7.0 (5.0, 9.0)	0.07
Total cost, CNY	11,463 (8,652, 14,858)	11,603 (9,368, 15,453)	0.10
Frailty, n (%)	67 (9.1)	26 (4.9)	0.01*
Cognitive impairment, n (%)	65 (8.8)	32 (6.0)	0.08
Anxiety/Depression, n (%)	34 (4.6)	73 (13.8)	<0.001**
Fracture, n (%)	72 (9.8)	52 (9.8)	1.00
Repeat hospitalization (6 m), n (%)	46 (6.3)	47 (8.9)	0.10
Medication count	5.0 (3.0, 7.0)	6.0 (4.0, 9.0)	<0.001**
Polypharmacy level, n (%)	​	​	<0.001**
<5 medications	353 (48.0)	137 (25.9)	​
5–9 medications	345 (46.9)	295 (55.8)	​
≥10 medications	37 (5.0)	97 (18.3)	​

Data are presented as median (interquartile range) for continuous variables and n (%) for categorical variables. Differences were analyzed using the Mann-Whitney U test and χ^2^ test, as appropriate. *P < 0.05, **P < 0.01. ADL, activities of daily living; CCI, charlson comorbidity index; PIM, potentially inappropriate medication.

### 3.2 Factors associated with PIM exposure

Table 2 presents the logistic regression analysis of factors associated with PIM exposure at discharge. In the multivariable model, higher medication count (OR 1.26, 95% CI 1.20–1.32, *P* < 0.001) and the presence of anxiety/depression (OR 2.27, 95% CI 1.43–3.61, *P* < 0.001) were significantly associated with an increased likelihood of PIM use. Conversely, higher CCI (OR 0.89, 95% CI 0.80–0.98, *P* = 0.02) and frailty (OR 0.51, 95% CI 0.29–0.89, *P* = 0.02) were independently associated with a lower likelihood of PIM use, suggesting a potentially more conservative prescribing approach for patients with severe comorbidities and frailty.

**TABLE 2:** Logistic regression analysis of factors associated with PIM exposure at discharge.

Variable	Univariable OR (95% CI)	*P* value	Multivariable OR (95% CI)	*P* value
Age, years	1.01 (0.99–1.02)	0.45	1.00 (0.99–1.02)	0.85
Sex (male)	0.89 (0.71–1.11)	0.29	0.85 (0.66–1.08)	0.18
ADL score	1.00 (0.99–1.00)	0.33	1.00 (0.99–1.00)	0.15
Diagnosis count	1.12 (1.06–1.18)	<0.001**	1.05 (0.97–1.14)	0.22
CCI	1.04 (0.96–1.12)	0.35	0.89 (0.80–0.98)	0.02*
Hospital length of stay, days	1.03 (1.00–1.07)	0.05*	1.01 (0.95–1.07)	0.80
Total cost (per 1,000)	1.02 (1.00–1.04)	0.04*	1.00 (0.97–1.03)	0.89
Critical condition	0.96 (0.74–1.23)	0.73	0.86 (0.62–1.19)	0.37
Medication count	1.26 (1.20–1.31)	<0.001**	1.26 (1.20–1.32)	<0.001**
Anxiety/Depression	3.30 (2.16–5.04)	<0.001**	2.27 (1.43–3.61)	<0.001**
Frailty	0.52 (0.32–0.82)	0.01	0.51 (0.29–0.89)	0.02*
Cognitive impairment	0.66 (0.43–1.03)	0.07	0.76 (0.47–1.24)	0.27
History of fracture	1.00 (0.69–1.46)	0.98	0.73 (0.48–1.11)	0.14

Univariable and multivariable logistic regression analyses were performed. Multivariable models were adjusted for age, sex, ADL, score; CCI, score, and other clinical covariates. *P < 0.05, **P < 0.01. ADL, activities of daily living; CCI, charlson comorbidity index; CI, confidence interval; OR, odds ratio; PIM, potentially inappropriate medication.

### 3.3 Association between PIM use and the 30-day composite outcome

**FIGURE 2:**
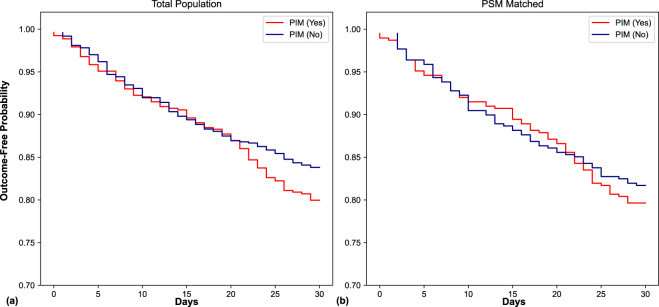
Kaplan–Meier survival curves for the 30-day composite outcome according to PIM status in the total population **(a)** and the PSM cohort **(b)**. The composite outcome includes unplanned readmission or emergency department visits. P values were calculated using the log-rank test (total population: P = 0.10; PSM cohort: P = 0.51). In both cohorts, no statistically significant difference in cumulative risk was observed between the PIM and non-PIM groups. PIM, potentially inappropriate medication; PSM, propensity score matching.

During the 30-day follow-up period, 225 composite events were recorded, with an event rate of 20.0% (106/529) in the PIM group and 16.2% (119/735) in the non-PIM group. Multivariable Cox regression analysis in the total population revealed that PIM use was associated with a trend toward an increased risk of the 30-day composite outcome, although this association did not reach statistical significance after adjusting for potential confounders (adjusted HR [AHR] 1.22, 95% CI 0.92–1.62, *P* = 0.16; Table 3). Kaplan-Meier analysis in the total population showed no significant difference (log-rank *P* = 0.10; Figure 2a).

**TABLE 3:** Association between PIM status and 30-day composite outcome in the total, PSM, and IPTW populations.

Population	Crude HR (95% CI)	*P* value	Adjusted HR (95% CI)	*P* value
Total population (N = 1,264)	1.25 (0.96–1.62)	0.09	1.22 (0.92–1.62)	0.16
PSM (N = 776, 388 pairs)	1.11 (0.81–1.54)	0.51	1.12 (0.81–1.54)	0.49
IPTW (N = 1,264)	1.25 (0.95–1.63)	0.09	1.26 (0.97–1.63)	0.09

Cox proportional hazards regression models were used to evaluate the association between PIM, status and 30-day composite outcome. Multivariable models were adjusted for age, sex, ADL, score; CCI, score, medication count, critical condition, and other baseline comorbidities. *P* < 0.05 was considered statistically significant. CI, confidence interval; HR, hazard ratio; IPTW, inverse probability of treatment weighting; PSM, propensity score matching.

### 3.4 PSM and robustness analysis

**FIGURE 3:**
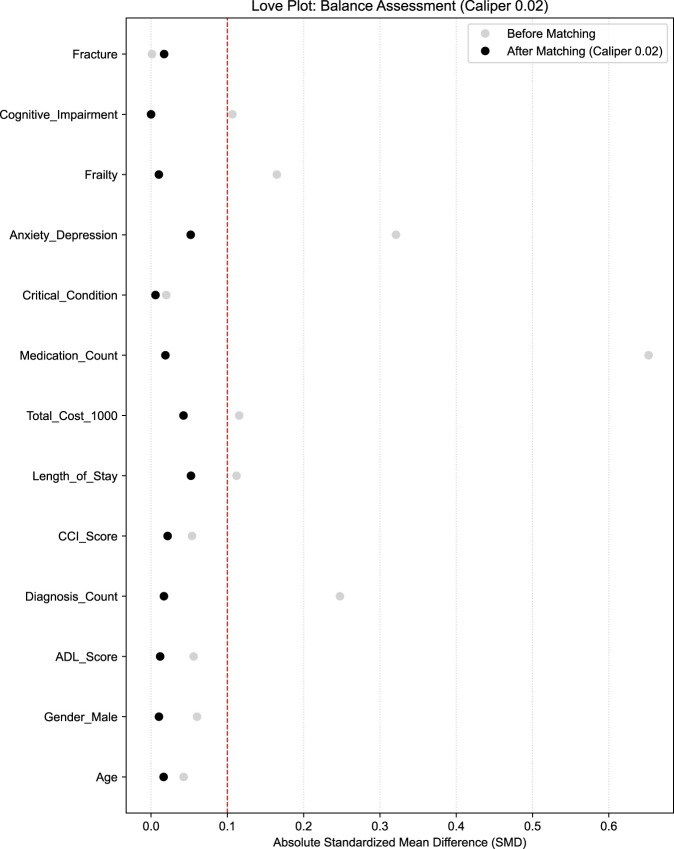
Love plot showing the SMDs of the covariates before and after PSM. After 1:1 matching, the SMDs of all covariates (including medication count and psychological status) were reduced to below the 0.1 threshold (dashed line), indicating a high degree of balance between the PIM and non-PIM groups. PIM, potentially inappropriate medication; PSM, propensity score matching; SMD, standardized mean difference.

To mitigate the effect of potential selection bias, 1:1 PSM with a 0.02 caliper was implemented, resulting in a well-balanced cohort of 388 pairs (N = 776). The Love plot (Figure 3) confirmed that all covariate SMDs were reduced to <0.1 after matching. In this matched cohort, the association between PIM status and the 30-day composite outcome remained non-significant (AHR 1.12, 95% CI 0.81–1.54, *P* = 0.49; Table 3). In the PSM cohort, Kaplan-Meier analysis also showed no significant difference (log-rank *P* = 0.51; Figure 2b). Sensitivity analysis with a stricter 0.01 caliper (352 pairs) further confirmed the robustness of these findings (AHR 1.06, *P* = 0.72).

The IPTW-adjusted HR for the 30-day composite outcome was 1.26 (95% CI 0.97–1.63, *P* = 0.09), consistent with the primary PSM findings. Covariate balance after IPTW was excellent, with all SMDs <0.01 and *P* > 0.80 (Supplementary Table S1).

### 3.5 Subgroup and drug-specific analyses

**FIGURE 4:**
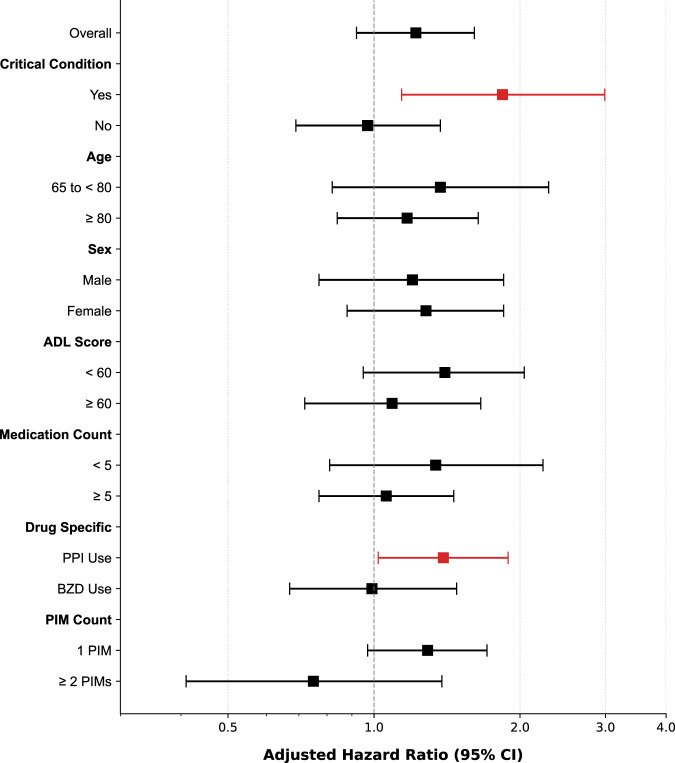
Forest plot of the AHRs for the association between PIM status and the 30-day composite outcome across clinical subgroups and for specific PIM subclasses. Notably, significant risk elevation was observed in critically ill patients (AHR 1.84, 95% CI 1.14–2.99) and for PPI-related PIM (AHR 1.39, 95% CI 1.02–1.89). AHR, adjusted hazard ratio; CI, confidence interval; PIM, potentially inappropriate medication; PPI, proton pump inhibitor.

Despite the non-significant overall effect of PIMs, subgroup analysis revealed potential heterogeneity in risk among subgroups. Among patients in the critical condition subgroup (reduced physiologic reserve), PIM exposure was associated with a nearly 2-fold increase in the 30-day composite outcome risk (AHR 1.84, 95% CI 1.14–2.99). The interaction *P* value for the critical condition subgroup was 0.07; therefore, this finding is considered exploratory. Regarding specific drug classes, PPI-related PIM was identified as a significant independent risk factor (AHR 1.39, 95% CI 1.02–1.89). A higher risk trend was also observed in patients with an ADL score <60 (AHR 1.40, 95% CI 0.95–2.04; Figure 4; Table 4).

**TABLE 4:** Subgroup and drug-specific analysis of the association between PIM status and the 30-day composite outcome.

Category	Subgroup	PIM+ (Event/N)	PIM− (Event/N)	Adjusted HR (95% CI)	*P* Value for interaction
Overall	All patients	106/529	119/735	1.22 (0.92–1.61)	–
Critical condition	Yes	41/135	37/194	1.84 (1.14–2.99)	0.07
​	No	65/394	82/541	0.97 (0.69–1.37)	​
Age	65 to <80	32/196	35/292	1.37 (0.82–2.29)	0.61
​	≥80	74/333	84/443	1.17 (0.84–1.64)	​
Sex	Male	46/231	54/343	1.20 (0.77–1.85)	0.93
​	Female	60/298	65/392	1.28 (0.88–1.85)	​
ADL score	<60	58/217	61/291	1.40 (0.95–2.04)	0.68
​	≥60	48/312	58/444	1.09 (0.72–1.66)	​
Medication count	<5	24/137	44/353	1.34 (0.81–2.23)	0.36
​	≥5	82/392	75/382	1.06 (0.77–1.46)	​
Drug-specific	PPI use	72/318	119/735	1.39 (1.02–1.89)	​
​	Benzodiazepine use	39/218	119/735	0.99 (0.67–1.48)	​
PIM count	1 PIM	92/431	119/735	1.29 (0.97–1.71)	​
​	≥2 PIMs	14/98	119/735	0.75 (0.41–1.38)	​

Subgroup analyses and interaction tests were performed using Cox proportional hazards regression models. Hazard ratios were adjusted for all potential confounders except the stratification variable. *P* for interaction was calculated to assess the heterogeneity of treatment effects across subgroups. For specific PIM, subclasses (PPI, and benzodiazepine), multivariable Cox regression models were performed with no PIM, as the reference, adjusting for all covariates. ADL, activities of daily living; CI, confidence interval; HR, hazard ratio; PIM, potentially inappropriate medication; PPI, proton pump inhibitor.

### 3.6 E-value assessment

E-values were calculated to assess the potential effect of unmeasured confounding on the significant findings. For the critical condition subgroup, the E-value for the point estimate was 3.08 (95% CI lower bound 1.54), indicating that a moderately strong unmeasured confounder would be required to negate the observed risk. For inappropriate PPI use, the E-value was 2.12 (95% CI lower bound 1.16). These results collectively support the robustness of the findings, particularly in the critical condition subgroup (Table 5).

**TABLE 5:** E-value analysis for significant associations to assess the effect of unmeasured confounding.

Association	Adjusted HR (95% CI)	E-value (point estimate)	E-value (lower 95% CI bound)
Critical condition: Yes	1.84 (1.14–2.99)	3.08	1.54
PPI use	1.39 (1.02–1.89)	2.12	1.16

E-value analysis was performed to assess the robustness of significant associations to unmeasured confounding. CI, confidence interval; HR, hazard ratio; PPI, proton pump inhibitor.

## 4 Discussion

In this study, using PSM and strictly controlling for key confounders, such as medication count and psychological comorbidities, overall PIM use was not an independent predictor of the 30-day composite outcome among hospitalized older patients. Our stratified analysis suggested potential heterogeneity in the 30-day composite outcome risk among patients taking PIMs. PIM use significantly elevated the risk of the 30-day composite outcome in the critically ill subgroup (reduced physiologic reserve) and among those with inappropriate PPI use, while no association was observed in non-critically ill patients; the test for interaction was not significant.

The finding that overall PIM use was not an independent predictor of the 30-day composite outcome among hospitalized older patients is consistent with the observations in the REPOSI cohort (Pasina et al., 2014), which demonstrated that after adjustment for clinical characteristics, PIMs showed no significant association with short-term adverse outcomes. Furthermore, Fabbietti et al. (2018) suggested that adverse clinical outcomes—such as hospital readmissions—that have conventionally been attributed to PIMs in institutionalized (Ruggiero et al., 2010) and community-dwelling (Cahir et al., 2014) older adults may actually be driven by polypharmacy. Fabbietti et al. (2018) showed that polypharmacy (defined as the use of >8 medications) was significantly associated with 3-month readmission (OR 2.72, 95% CI 1.48–4.99), whereas the presence of PIMs—defined by either the Beers or STOPP criteria—lost its significant association with readmissions when evaluated concurrently in the adjusted models. The authors concluded that the effect of polypharmacy on readmission was independent of the use of PIMs. Similarly, Basnet et al. (2018) reported that an increased medication count was significantly associated with 30-day hospital readmission, while no significant association was observed for PIMs after controlling for total medication count. A recent cohort study by Sombut et al. (2025) demonstrated that while PIMs were highly prevalent at discharge, they were not independently associated with early unplanned readmissions in multivariable survival models. Rather, overarching factors, such as polypharmacy and comorbidity burden, were the actual drivers of post-discharge outcomes. Therefore, when the medication count is strictly controlled, as in the present study, the generic PIM label may lose its independent predictive value for readmissions (Fabbietti et al., 2018).

Although no statistically significant association was observed between PIM use and 30-day readmission in the overall population in the present study, this should not be interpreted as suggesting that PIMs are harmless. The observed HR was 1.22, with the upper bound of the 95% CI reaching 1.62. This suggests that while we cannot reject the null hypothesis statistically, potential adverse effects in the general population cannot be entirely ruled out. Therefore, the effect size of PIM risk in the general older population may be small, but it may be easily magnified or inflated by confounders, such as polypharmacy, which were not fully adjusted previously (Degen and Schottker, 2025; Fabbietti et al., 2018). Consequently, rather than debating the faint and uncertain risk signals in the general population, clinical intervention should prioritize high-risk groups (e.g., critically ill patients, patients taking PPIs).

The most prominent methodological strength of this study lies in the use of PSM to determine confounding by indication—a persistent challenge that has plagued research in this field (Kyriacou and Lewis, 2016; Degen and Schottker, 2025). Pre-matching data revealed that the PIM group had a significantly higher medication count (6.0 vs. 5.0) and a more prevalent diagnosis of anxiety/depression (13.8% vs. 4.6%), compared with the non-PIM group. If only crude adjustments were to be performed, these characteristics—representing clinical complexity and intensity of medical need—are often misinterpreted as intrinsic toxicity of the medications themselves (Fabbietti et al., 2018). In the present study, by achieving a near-perfect balance in medication count (with the SMD decreasing from 0.65 to 0.02), the crude risk association (crude HR 1.25) disappeared. This finding elucidates contradictions in the existing literature. For instance, while Weir et al. (2020) observed adverse clinical outcomes with PIM use (primarily concentrated in new prescriptions), our results align more closely with high-quality evidence from Fabbietti et al. (2018) and Pasina et al. (2014), suggesting that after strictly controlling for medication count as a core confounder, the isolated PIM label no longer independently predicted short-term readmission risk (AHR 1.12, *P* = 0.49). Furthermore, our findings are highly consistent with the recent study by Sombut et al. (2025), who showed no significant association between PIM use and early unplanned readmissions after adjusting for comorbidity index and hospital LOS. That study highlighted that comorbidity burden and polypharmacy are true significant predictors of readmission. A key distinction is that while Sombut et al.’s population was derived from multiple departments at a tertiary hospital with high heterogeneity, the present study focused exclusively on the geriatrics department, where patients exhibited a more consistent frailty baseline and more complex medication regimens (average of eight medications in the PIM group). In summary, by strictly determining the influence of medication count via PSM, our data confirm that PIM use is primarily a marker of clinical complexity and polypharmacy, rather than a direct marker of short-term readmission risk (Fabbietti et al., 2018).

Our stratified analysis suggested potential heterogeneity in the 30-day composite outcome risk among patients taking PIMs. The risk of the 30-day composite outcome associated with PIM use was primarily observed in the critically ill subgroup (reduced physiologic reserve) and among patients with PPI-related PIM, while no association was observed in non-critically ill patients. Specifically, while PIM use significantly increased the 30-day risk in critically ill patients (HR 1.84), it showed no such effect in non-critically ill patients (HR 0.97). Physiologic reserve is a multidimensional indicator of resilience to biological stresses, indicating one’s ability to maintain homeostasis across organ systems (Hu et al., 2024; Saloner et al., 2022; Whitson et al., 2015). Reduced physiologic reserve reduces the threshold for drug tolerance and increases the risk of PIM-related adverse outcomes in older adults (Corsonello et al., 2010; McLean and Le Couteur, 2004). Therefore, when critically ill patients—who already have exhausted physiologic reserve—are exposed to PIMs, their compromised homeostatic mechanisms may fail to buffer pharmacological toxicity, leading to adverse outcomes, as observed in the present study.

The surge in PIM-related risk within the critically ill subgroup (HR 1.84, 95% CI 1.14–2.99) represents a key finding of this study, providing evidence for the physiologic reserve theory in geriatric medicine. Although patients with a critical condition inherently possess a higher baseline readmission risk, our study used PSM to observe the independent effects of PIMs within subgroups of equivalent clinical severity. This “drug–disease interaction” suggests that medication safety assessments in vulnerable populations should not be decoupled from the patient’s overall physiological context. Older patients who have undergone intensive care or remain in a critical state possess a homeostatic reserve that is on the verge of exhaustion (Hilmer et al., 2019). Consequently, any additional PIM burden is no longer merely a potential risk, but it becomes a critical trigger precipitating acute clinical decompensation (Oshima Lee and Emanuel, 2013). For instance, in critically ill patients with prolonged bed rest and impaired immune defenses, PPI-induced disruption of the gastric acid barrier may increase the risk of HAP or *Clostridioides difficile* infection, potentially contributing to clinical deterioration and readmission (Nalabothula et al., 2025). This suggests that for fragile phenotypes with extremely low physiologic reserve, the therapeutic window of medications is drastically compressed. Therefore, designating such patients as the highest priority population for pre-discharge medication reconciliation and precision deprescribing (individualized, data-driven reduction or discontinuation of medications that are no longer beneficial, are inappropriate, or are causing adverse effects or healthcare resource utilization) (Scott et al., 2015) offers the most definitive clinical benefit. Although this study is limited by its retrospective design and did not directly measure biomarkers of physiologic reserve, using critical status as a clinical phenotypic surrogate for depleted physiologic reserve is pathophysiologically sound. The significant 30-day composite outcome risk elevation observed in the critical condition subgroup (HR 1.84) supports physiologic reserve as an important consideration in drug safety evaluation.

PPI-related PIM use also demonstrated a higher risk (HR 1.39) of the 30-day composite outcome, while users of other PIMs, namely benzodiazepines, did not (HR 0.99). A recent study also reported the increased risks associated with potentially inappropriate PPI use, showing a 60% higher risk of 1-year mortality and a 27% increased risk of 1-year rehospitalization (Elli et al., 2025). From a biological perspective, chronic acid suppression by PPIs impairs the gastric acid barrier, inducing bacterial overgrowth and increasing the risk of HAP or *Clostridioides difficile* infection via micro-aspiration (Nalabothula et al., 2025; Wu et al., 2025). As highlighted by Blackett et al. (2021), prophylactic PPIs initiated during hospitalization are often inadvertently continued at discharge; therefore, combating PPI overuse driven by clinical inertia at this critical interface of care is paramount. Conversely, although benzodiazepines were prevalent in the PIM group, they did not demonstrate a significant 30-day composite outcome risk (HR 0.99, 95% CI 0.67–1.48). This may reflect the low incidence of acute events such as falls or fractures in the short term, or the presence of “channeling bias”—a phenomenon where clinicians tend to avoid prescribing these medications to the frailest patients or those with a history of falls, thereby statistically diluting the observed risk (Acton et al., 2023).

Several methodological considerations should be noted. First, the overall association between PIM use and the composite outcome was non-significant, and the interaction test for the critical condition subgroup did not reach statistical significance (P = 0.07). Therefore, the positive findings in critically ill patients should be viewed as exploratory rather than confirmatory. Second, the analysis of specific drug classes (PPI-related PIM) was one of several exploratory analyses, so a type I error due to multiple comparisons cannot be excluded. Third, the small sample size in some subgroups (e.g., ≥2 PIMs, n = 98) limited statistical power, which may have contributed to the non-significant interaction and the lack of a dose-response effect. These findings should be considered hypothesis-generating for future studies.

We did not observe a trend of increasing risk with an increasing number of PIMs. This is likely attributable to the relatively small sample of patients with ≥2 PIMs (n = 98), which restricted the statistical power, rather than representing a “protective” effect. Additionally, patients with a highly complex medication burden may inherently receive stricter adherence management and more intensive medical surveillance. Such intensified medical surveillance may partially offset the intrinsic toxicity of the medications (Weir et al., 2020). Furthermore, it is plausible that those who tolerate multiple PIMs and remain stable for discharge may have better physiological reserve, although this remains speculative. In contrast, fragile individuals susceptible to ADEs may have discontinued therapy after exposure to a single PIM owing to intolerance—a phenomenon known as the “depletion of susceptibles” effect (Acton et al., 2023). Consequently, the results suggest that a simple PIM count is inadequate for a linear extrapolation of clinical risk. Clinical decision making should prioritize the specific drug classes involved and their interaction with the patient’s individual physiological phenotype.

### 4.1 Future perspectives

Overall, the clinical risk associated with PIMs was not uniformly distributed across the older population; instead, it was primarily observed in patients with a critical condition (depleted physiologic reserve) and those with inappropriate PPI use. Therefore, clinical practice should move beyond broad, checklist-based screening, toward precision deprescribing strategies (Scott et al., 2015) that are centered on specific patient clinical phenotypes and high-risk drug categories. Prioritizing limited clinical pharmacy resources for critically ill patients and inappropriate PPI prescriptions, rather than applying uniform intervention intensity to all PIMs, represents an optimal pathway for reducing readmission rates and optimizing healthcare resource allocation. Practical implementation could involve integrating the risk phenotypes identified in this study into Clinical Decision Support modules within the Electronic Medical Record systems (Scott et al., 2015). For instance, when the system detects a critical condition tag or a PPI prescription, a mandatory medication review alert could be automatically triggered at the discharge order interface (Elder et al., 2026). Such a strategy would drive the evolution of clinical pharmacy services from low-efficiency blanket screening to high-yield targeted intervention, thereby maximizing the efficacy of readmission prevention without increasing labor costs.

### 4.2 Limitations

Several limitations of this study warrant consideration. First, the retrospective, single-center design precludes the complete elimination of unmeasured confounding factors, such as social support (e.g., living alone) and objective nutritional biomarkers (Glans et al., 2020). Second, the 30-day observation window may have underestimated the long-term risks associated with PIMs, such as cognitive decline. Similarly, the inability to determine the duration of PPI use and the use of an indication-based assessment may have led to misclassification of short-term PPI use or failure to capture cases of appropriate indication with excessively long-term use. Third, 30-day mortality was not included in the composite outcome, which may have led to risk underestimation in the most fragile patients and may limit the ability to account for competing risks. Nevertheless, given that the composite outcome included ED visits and readmissions, most serious adverse events are considered to have been captured. Fourth, the lack of post-discharge medication adherence data constitutes a critical limitation. Given the high polypharmacy rate in the PIM group (6.0 vs. 5.0 medications), patients may have engaged in “protective non-adherence” (self-reducing PIM intake), which could have led to underestimation of the actual biological toxicity of PIMs (Poorcheraghi et al., 2023). Finally, the subgroup analyses were exploratory rather than confirmatory, and they may be limited by multiple comparisons or restricted statistical power.

## 5 Conclusion

After rigorously controlling for the confounding effects of polypharmacy, the generic PIM label lost its independent predictive value for the 30-day composite outcome. The clinical risk associated with PIM use was not uniformly distributed across the older population; rather, it was primarily observed in critically ill patients (reduced physiologic reserve) and those with inappropriate PPI use. Clinical interventions should prioritize these high-risk phenotypes for precision deprescribing.

## Data Availability

The datasets presented in this article are not readily available because the original de-identified datasets are available from the corresponding authors upon reasonable request. Requests to access the datasets should be directed to zengzhen412@sohu.com.
