# Supplementary material for: Association between potentially inappropriate medications and 30-day outcomes among hospitalized older adults with reduced physiologic reserve: a retrospective cohort study
Source: Front Pharmacol. 2026 May 20;17:1813958. doi: 10.3389/fphar.2026.1813958 (PMC13229643; doi:10.3389/fphar.2026.1813958)
Supplement: Supplementary file 1 [file DataSheet1.PDF]

Supplementary Material

1 Supplementary Table

Supplementary Table S1. Baseline Characteristics and Covariate Balance Before and After IPTW

**Characteristic**	**Before IPTW (N = 1,264)**		**After IPTW (N = 1,264)**	
	**SMD**	***P* value**	**SMD**	***P* value**
Age	0.05	0.59	0.01	0.92
Sex (Male)	0.06	0.32	0.01	0.89
ADL score	0.06	0.30	0.01	0.90
CCI	0.05	0.35	<0.01	0.98
Medication count	0.41	<0.001	0.01	0.94
Critical condition	0.02	0.78	0.01	0.91
Anxiety/Depression	0.32	<0.001	0.01	0.85
Frailty	0.17	0.01	0.01	0.90
Cognitive impairment	0.11	0.08	0.01	0.93
History of fracture	<0.01	1.00	<0.01	0.99

Standardized mean differences (SMD) were used to evaluate covariate balance before and after IPTW. SMD < 0.10 was considered adequate balance. ADL, activities of daily living; CCI, Charlson Comorbidity Index; IPTW, inverse probability of treatment weighting; SMD, standardized mean difference.
